# A Conversation with Leslie Schoop

**DOI:** 10.1021/acscentsci.4c00239

**Published:** 2024-02-27

**Authors:** Sam Lemonick

Room-temperature superconductors lie somewhere near the boundary of science fact and science fiction. These hypothetical materials with zero electrical resistance could enable more powerful computers and help solve global energy problems. But while room-temperature superconducting materials are theoretically possible, no one has made one or definitively predicted one.

**Figure:**
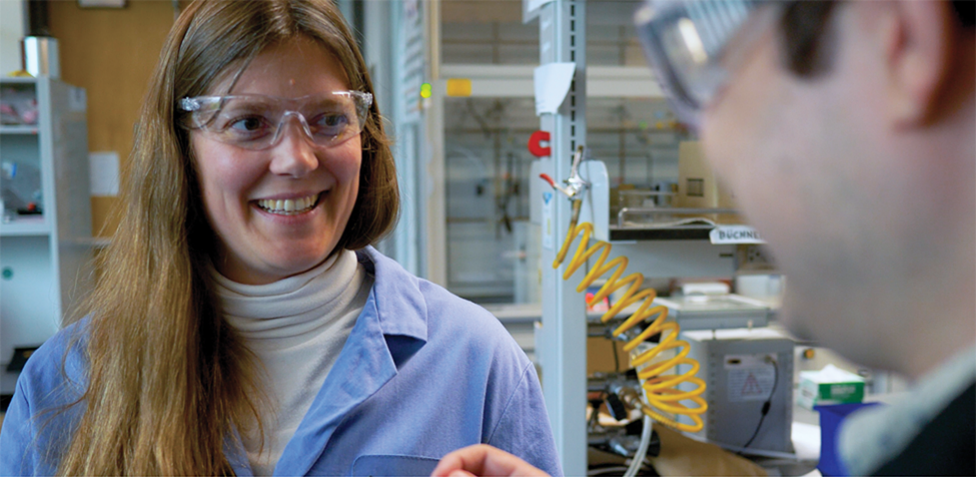
Credit: Leslie Schoop/Sean Schools

Still, every few years or so, new claims emerge of a room-temperature superconductor. It happened again in July 2023 when a group of researchers unveiled a preprint—an article published before peer review—describing LK-99, a copper-doped lead oxide phosphate that displayed superconductivity at temperatures as high as 127 °C. But the frenzy over LK-99 spread wider and reached a higher pitch than previous reports did. After rounds of claims and counterclaims, the scientific community determined that the tantalizing properties observed in samples of the material were not a result of superconductivity. Instead, magnetism stemming from impurities in the LK-99 synthesis was to blame.

Leslie Schoop, a solid-state chemist at Princeton University, was among the prominent voices challenging predictions about LK-99 on X (formerly Twitter) and later in the scientific literature.

Sam Lemonick spoke with Schoop about her role in debunking claims about LK-99, the future of room-temperature superconductors, and how scientists respond to exciting scientific results. This interview was edited for length and clarity.

## What do you remember about July 2023, when the first claims that LK-99 was a room-temperature superconductor emerged and started getting a lot of attention on social media and elsewhere?

Lots of people texted me about it. Initially, I really ignored it because room-temperature superconductivity comes up every couple of years. This was the first one that got so much traction.

I was actually sick when it really started getting interesting. I was just pretty disastrous in my bed, and I kept getting texts about it. So I started reading a bit more, and then I realized that because of this hype, at some point I will want to answer the question for myself.

## How did you first go about answering the question of whether LK-99 was a room-temperature superconductor?

I asked if there was a volunteer in my group who would just put a sample in a furnace. I didn’t have a lot of investment, but it was like, let’s just cook one. It’s easy enough—the recipe is right there. And let’s just see for ourselves what it is. I had one student [graduate student Scott Lee] who was interested in doing it.

Then I had to fly to California. There was a meeting with other scientists, and lots of people talked about it. I talked about it a lot too. I wouldn’t have told a journalist there was zero chance that [LK-99 is a superconductor], but privately, I’m telling you it’s not a thing. But some others weren’t so sure. And I thought, “These are scientists I really respect, so maybe I’m being too harsh.”

I kept following up with my student. We had a sample, but there were transparent crystals in there. [The original claims about LK-99 showed photos of an opaque material. The difference in appearance suggested that the original material had impurities, which would later prove to be the source of its initially intriguing properties—Ed.] So it was very clear that LK-99 is not a superconductor, but I still didn’t think that we should publish anything about it. I thought, “Well maybe those samples are different, and let’s just see how things play out.”

**Figure:**
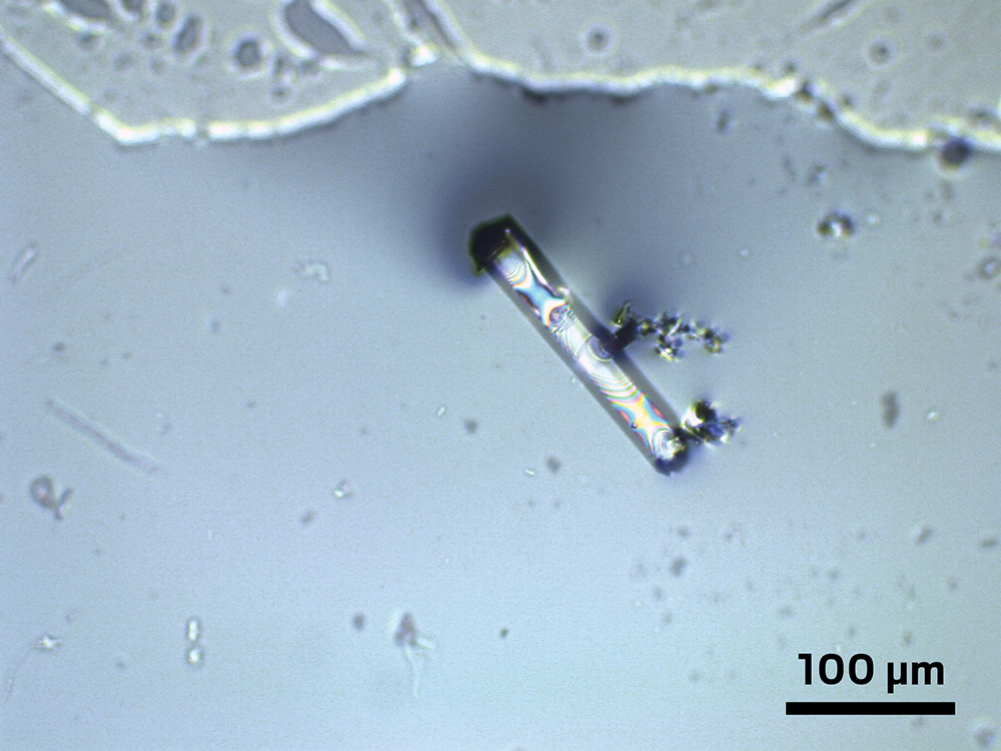
A transparent crystal of pure LK-99 made by Leslie Schoop’s graduate student Scott Lee. Credit: Scott Lee.

## What made you change your mind about joining the public discussion over LK-99?

The moment when I got really motivated to do something was when several studies using density functional theory (DFT) [a widely used computational chemistry technique that can model atoms, molecules, and materials] came on to *arXiv*. And some of them were hyped on Twitter; others were not. They were claiming to have found a DFT-based reason for the superconductivity.

So for half my life, I have called out the incorrect use of DFT in solids. You have to be pretty careful because there are significant shortcomings with them. The structure [of LK-99] wasn’t really known yet, right? They just assumed the structure based on [the original] preprint. And if you don’t put the correct structure in your DFT calculation, you’re not going to get the correct electronic structure. I have actually given lectures at summer schools before where I have called out misuses of DFT.

And then I remember there was this call with 3,000 people in it discussing the theory behind LK-99. And I just felt like, now this is too far. This is when I started voicing criticism on social media.

I was, like, OK, that’s what I trained for my entire life.

## Looking back, what do you think about the way LK-99’s story played out—about how public the scientific debate over superconductivity was?

I mostly liked the discourse about LK-99. Yes, there was hype, but it was not really ugly—everybody was excited and was very reasonable in their comments. So I think in general it was positive.

The frenzy, it’s not like something that never happens, right? Sometimes everybody gets into a frenzy when something new crops up. I think that’s normal. And mistakes always happen, and then they [get corrected] later. I think nobody was doing fraud there. They openly showed their data.

Despite the hype and the negative aspects of this, I think it was a pretty positive experience that people get so excited about physics or solid-state chemistry. At some point, I was making Twitter threads about flat bands and phonons. People asked me about this on Twitter. I don’t even talk about phonons in my graduate course.

## Do you think someone will find a room-temperature superconductor eventually?

We should definitely not dismiss any claim of it. We don’t have a theory saying it’s not possible. Superconductivity so far has always surprised us. Very few superconductors have been predicted before they were found. So if there’s a room-temperature superconductor, it’s probably going to surprise us.

It would be cool if they found one. I really hope they do.

## Sam Lemonick is a freelance contributor to

Chemical & Engineering News, *the independent news outlet of the American Chemical Society.*

